# Dietary and lifestyle behavior in adults with epilepsy needs improvement: a case-control study from northeastern Poland

**DOI:** 10.1186/s12937-021-00704-6

**Published:** 2021-06-29

**Authors:** Kamila Szałwińska, Monika Cyuńczyk, Jan Kochanowicz, Anna M. Witkowska

**Affiliations:** 1 Department of Food Biotechnology, Medical University of Białystok, Białystok, Poland; 2 Department of Neurology, Medical University of Białystok, Białystok, Poland

**Keywords:** Epilepsy, Diet, Nutritional status, Physical activity, Lipid profile, Vitamin D

## Abstract

**Background:**

Several factors predispose individuals with epilepsy to chronic diseases. Among them, nutrition and lifestyle factors have not been sufficiently studied. Therefore, the aim of this study was to evaluate patients with epilepsy in terms of diet, body composition and physical activity compared to healthy sex- and age-matched subjects to investigate whether there are risk factors for nutritional deficiencies and risk factors for the development of metabolic diseases.

**Methods:**

The case-control study involved 60 epileptic male and female volunteers and 70 healthy controls matched according to age and sex. Medical information was collected during the study, and a detailed questionnaire regarding eating and lifestyle habits was conducted. Physical activity was evaluated using the International Physical Activity Questionnaire (IPAQ). Nutritional status was assessed by bioelectric impedance. Venous blood samples were taken for lipid and 25-hydroxyvitamin D3 (25(OH)D3) analyses.

**Results:**

A tendency toward an increase in LDL cholesterol was found in the individuals with epilepsy. Significantly higher body fat and insignificantly higher visceral fat were found in epileptic men than in healthy men. In epileptic women, a tendency toward a lower lean body mass was found. Patients with epilepsy were more sedentary, consumed less cottage cheese, fruit, pulses, nuts and seeds, vitamin C and potassium, and consumed more sugar-sweetened soda, fat and sodium than healthy people. On a positive note, individuals with epilepsy consumed less coffee and alcoholic beverages. More than 80% of the epileptic volunteers had diets that were low in folic acid, vitamin D and calcium, but a similar tendency was observed in the healthy volunteers. A higher percentage of the patients with epilepsy had diets that were low in niacin, vitamin C and potassium than the control group (25% vs. 7, 50% vs. 31% and 73 vs. 56%, respectively). A significantly lower serum concentration of 25(OH)D3 was observed in epileptic individuals and was found to be positively modulated by physical activity.

**Conclusions:**

The results indicate that several behavior-related habits, which may predispose epileptic people to cardiovascular disease, need to be improved. For this reason, patients with epilepsy should be provided with more comprehensive medical care, including advice on nutrition and physical activity.

**Supplementary Information:**

The online version contains supplementary material available at 10.1186/s12937-021-00704-6.

## Background

Epilepsy is a chronic disease of the nervous system caused by structural, genetic, metabolic, infectious, or immunological factors that affects people of all ages [1]. In many cases, the cause of the disease is unknown. Sixty percent of epilepsy cases are idiopathic and have no clear cause, but a genetic burden is possible [2].

In epilepsy, deficiencies in nutrients such as B vitamins, vitamin D, zinc, and selenium have been observed [3–6]. The most common consequence of vitamin D deficiency is the loss of bone mass, which can occur in epilepsy as a result of pharmacotherapy, including the direct effect of drugs on bone tissue [7, 8]. An important problem in epilepsy is the co-occurrence of diet-related disorders. The epileptic population is characterized by a higher risk of cardiovascular disease (CVD) and dyslipidemia, which may be a consequence of behavioral risk factors, medication, or seizure-related cardiac arrythmias [9–11]. In addition, epileptic patients tend to have excessive body weight and abdominal obesity, which are risk factors for metabolic diseases [12]. Despite some controversy, it is believed that vitamin D may be important for CVD prevention [13, 14].

The role of physical activity in epilepsy has been discussed by medical specialists, caregivers, and patients themselves over time. Recommendations and guidelines issued by medical organizations are few and general, although in recent years, they have been aimed at encouraging rather than reducing physical activity in this group of patients [15, 16]. Although the possibility of practicing particular types of exercises and exercise intensities should always be considered individually with regard to factors such as the frequency of seizures or the type of epilepsy, there are studies showing that physical activity can have a positive effect on the course of the disease [16]. Patients with epilepsy often limit their physical activity mainly due to a fear of seizures and are less physically fit than healthy individuals [17, 18].

Some irregularities in the dietary habits, nutritional status, and lifestyle of the adult epileptic population have been identified over time, but there is limited knowledge in this area, motivating further research. Therefore, the aim of this study was to evaluate patients with epilepsy in terms of diet, body composition and physical activity compared to healthy sex- and age-matched subjects to investigate whether there are risk factors for nutritional deficiencies and risk factors for the development of metabolic diseases. Research methods in this study included anthropometric measurements (body weight, height, body mass index, body composition by bioelectrical impedance), dietary assessment (3-day 24-h dietary records, food frequency questionnaire), and biochemical analyses (serum lipid and vitamin D levels). Physical activity was assessed using the International Physical Activity Questionnaire (IPAQ).

## Methods

### Design and study groups

**Fig. 1:**
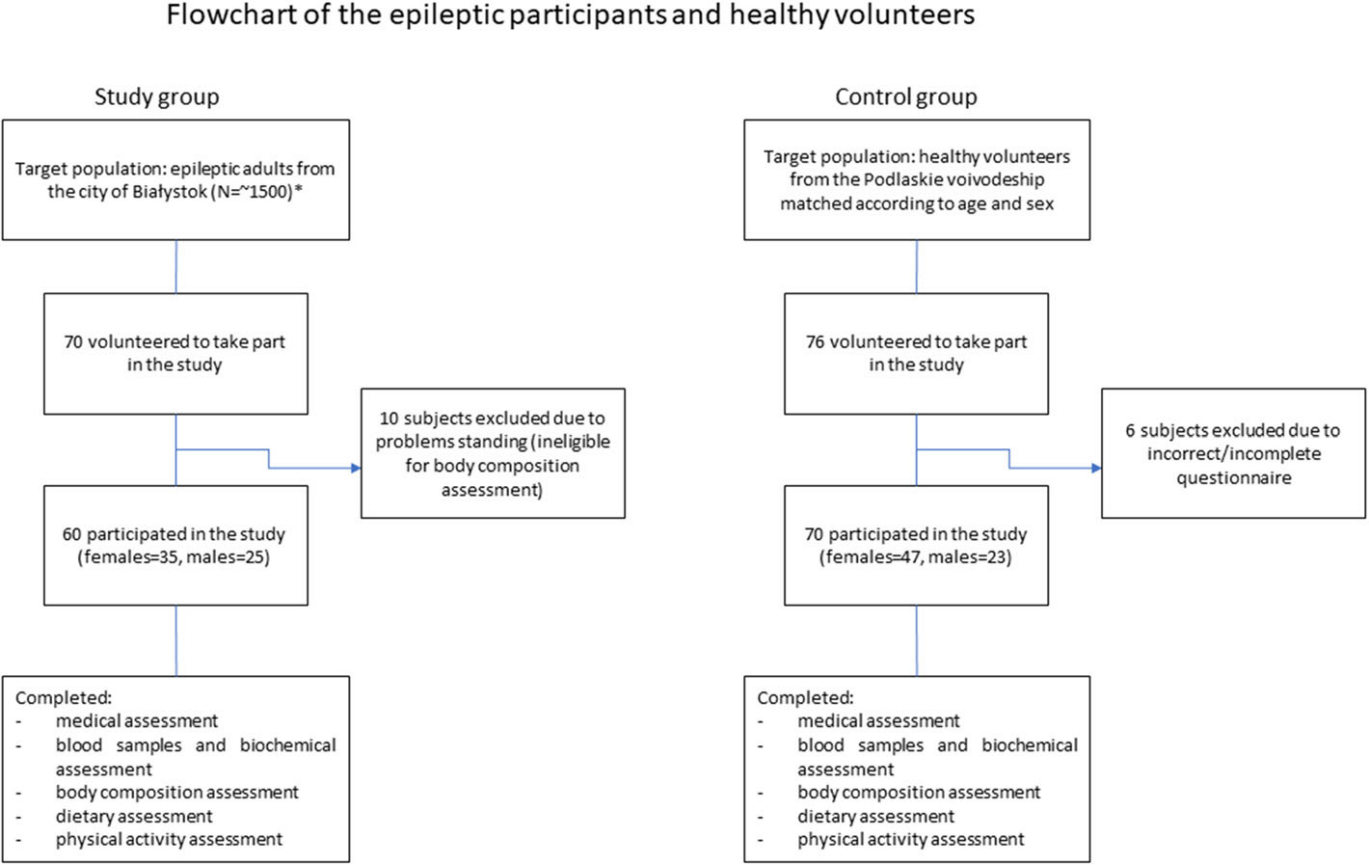
Flowchart of the epileptic and healthy participants. *Calculations were performed according to the Register of Statistics Poland (total estimated number of epilepsy cases minus children with epilepsy)

The study was conducted from 2016 to 2019 at Kendron neurological clinic in Białystok. The eligibility criteria were as follows: adult patients who had suffered from epilepsy for at least 1 year. The recruitment process was conducted on a continuous basis through the Kendron neurological clinic, media advertisements (press, television and internet), and health center advertisements aimed at epileptic patients from the city of Białystok. During the study, mentally ill patients were supported by caregivers. Of the 70 patients who volunteered to participate in the study, 10 had incomplete records due to inability to take part in the body composition analysis (problems with maintaining a standing position). Eventually, the study group included 60 epileptic patients aged 18–73 years (mean age 37.22 ± 12.88). A flowchart of the epileptic participants is provided in Fig. 1.

The control group consisted of 70 healthy subjects matched according to age and sex. The detailed characteristics of the study and control groups are presented in Table 1. Medical information about the cause of the disease, its onset, duration, symptoms, treatment, and mental comorbidities was obtained from patient medical records. The medication used in the form of monotherapy included carbamazepine, valproic acid, lamotrigine, levetiracetam, and pregabalin. In the case of polytherapy, patients took the abovementioned medication in different combinations with less frequently used drugs, including topiramate and gabapentin. The diseases and mental disorders associated with epilepsy included depression, anxiety, and schizophrenia.

**Table 1:** Characteristics of the study group vs. a healthy control

	Study Group ***N*** = 60	Healthy Control ***N*** = 70	***P*** value
General Characteristics			
Gender (%)			
Women	58.3	67.1	0.299
Men	41.7	32.9	
Age (years) mean ± SD	37.22 ± 12.88	37.13 ± 11.65	0.885
Occupation (%)			
Retirement/pension	36.7	2.9	< 0.001
Parental leave	0	2.9	
Unemployed	10	1.4	
Part-time job	8.3	7.1	
Permanent employment	36.7	82.9	
Student	8.3	2.9	
BMI [kg/m2] (%)			
Underweight (BMI < 18.5)	1.7	1.4	0.935
Normal weight (BMI 18.5–24.99)	45	45.7	
Overweight (BMI 25–29.99)	36.7	37.1	
Obesity (BMI > 30)	16.7	15.7	
Vitamin D supplementation (%)	11.7	7.1	0.378
**Characteristics of the Study Group**			
Age at disease onset (years) mean ± SD	18.25 ± 13.38	–	–
Duration of the disease (years) mean ± SD	18.98 ± 15.63	–	–
Number of medications taken mean ± SD	1.4 ± 0.7	–	–
Type of epilepsy (%)			
Idiopathic	73.3		–
Post-neonatal	6.7		
Post-traumatic	13.3	–	
Post-operative	6.7		
Seizures (%)			
Yes	43.4		–
No	56.6	–	
Anti-epileptic medication			
Monotherapy	53.3	–	–
Polytherapy	46.7		
Mental illnesses and disorders (%)	23.3	–	–

*N* number, *SD* standard deviation, *BMI* Body Mass Index

Data on age, employment, body measurements and vitamin D supplementation were also collected.

Body measurements such as height and body mass were obtained by personnel trained in standard procedures. Body mass index (BMI) was calculated as weight in kilograms divided by the square of height in meters.

STROBE guidelines for case-control research were followed in designing and reporting this study.

### Blood sample collection and biochemical analyses

7Twelve milliliters of venous blood from the antecubital vein was collected from the participants after fasting. The samples were left to clot for 30 min. Blood samples were centrifuged at 1500 x g for 10 min, and the serum was separated immediately after centrifugation. The obtained serum was frozen to − 80 °C and stored until subsequent determination. The lipid profile (total cholesterol, LDL and HDL cholesterol, and triglycerides) was determined using standard laboratory procedures at the University Clinical Hospital, Department of Biochemical Diagnostics of the Medical University of Białystok, which is accredited for performing laboratory tests.

### Body composition assessment

Body composition was assessed using the bioelectric impedance method using the InBody 270 analyzer (InBody Co. Ltd., Seoul, Korea). The electrical resistance was measured, which depends on both resistance and reactance. Resistance refers to the resistance of individual tissues, while reactance refers to the electrical capacity of cell membranes that act as capacitors [19]. Body mass (BM), lean body mass (LBM), percentage body fat (PBF), skeletal muscle mass (SMM), and percentage water content (PWC) were determined in both the epileptic and control subjects.

### Dietary assessment

After consenting to participate in the survey, a detailed questionnaire concerning eating and lifestyle habits was completed by the epileptic and control participants. In the case of mentally disabled epileptic participants, the questionnaire was completed by the patient’s caregiver. Food consumption frequency and 24-h dietary records for the three consecutive days preceding the study were recorded by a certified dietician (K. Szałwińska) trained in methods of dietary assessment. The questionnaire regarding the habitual frequency of food consumption, which was based on the National HES Manual, Poland [20], included 6 types of frequencies ranked from lowest to highest: 1: usual consumption once a month or less often; 2: consumption 2–4 times a month; 3: consumption 3 times a week; 4: consumption 4–6 times a week; 5: consumption daily; and 6: consumption several times a day. The data obtained from 3-day 24-h dietary recalls were compiled using the computer program Diet 5.0 (Food and Nutrition Institute, Warsaw), and energy and nutrients were calculated. The results were compared with the current nutrition recommendations for the Polish population [21].

### Determination of 25-hydroxyvitamin D3

The serum concentration of 25-hydroxyvitamin D3 (25-(OH)D3) was determined by high-performance liquid chromatography (HPLC) using a Prominence system (Shimadzu, Kyoto, Japan) consisting of an LC-20 AD solvent delivery system, a DGU-20A5 degasser, a ThermaSphere TS-130 column heater (Phenomenex, Torrance, CA, USA), and an SPD-M20A diode-array detector (DAD). Chromatographic separations were performed on a Synergi Hydro-RP 80 Å (250 × 4.6 mm) 4 μm column.

A 0.35 mL mixture of methanol-2-propanol (80:20 v/v) was added to 0.5 mL of serum. The samples were vortexed for 30 s and subsequently extracted three times with 2 mL of hexane. The extraction procedure consisted of mixing with n-hexane containing 0.01% butylated hydroxytoluene (BHT) for 60 s on a vortex mixer and transferring the supernatant to glass test tubes. Phases were separated by centrifugation for 10 min at RCF 2100 x g, and the upper phases were collected and dried under nitrogen at room temperature. The residue was dissolved in 100 μL of a mobile phase of acetonitrile and methanol (75:25 v/v), and 50 μL was injected. Isocratic separation was performed in a 30-min run with a flow rate of 1.5 mL/min with the column heater temperature set to 25 °C. The 25(OH)D3 concentration was determined at 265 nm. A calibration curve was prepared using four concentrations of 25(OH)D3 (Sigma Aldrich, Saint Louis, MN, USA) in the range 37.5–300 nmol/l. The recovery was 83%. The interpretation of the results included the season of the year in which the blood samples were taken.

Due to differences in sunlight at different times of the year, the results were adjusted for the spring-summer and autumn-winter periods.

### Physical activity assessment

The International Physical Activity Questionnaire (IPAQ) short form, the Polish language validated version, was used to assess the level of physical activity in epileptic and control participants [22]. The level of physical activity was calculated by assessing the intensity of physical activity during the 7 days prior to the survey and the time spent walking and sitting. Physical activity was expressed in metabolic equivalents of task MET-min/week, which is an equivalent of basal metabolic rate equal to energy expenditure, which corresponds to 3.5 mL O_2_ per kilogram of body weight per minute [23]. Taking this into account, physical activity is classified into one of three activity levels: 1) high (3 or more days of intense physical activity with at least 1500 MET-min/week or 7 or more days of any combination of physical activity exceeding 3000 MET-min/week), 2) moderate (3 or more days of intense physical activity with no less than 20 min per day, 5 or more days of moderate physical activity or walking of no less than 30 min per day, or 5 or more days of any combination of physical activity exceeding 600 MET-min/week), or 3) low (no physical activity at all or no sufficient or high level activity).

### Statistical analysis

The results were analyzed using the IBM SPSS Statistics package, IBM Corp., New York, USA. In group comparisons of quantitative data, Student’s t-test was used. Pearson’s Chi^2^ test of independence was used to compare the categorical variables between the groups. The relationships between the examined parameters were determined using the Spearman correlation coefficient. To compare the level of 25(OH)D3 in blood serum, statistical weights were used, which took into account two seasons differing in sunshine intensity in Poland, spring-summer (April–September) and autumn-winter (October–March) periods. For the calculations, *p* < 0.05 was assumed to be statistically significant.

## Results

In this study, 60 epileptic individuals were eligible to participate. Of them, 23.3% had a mental illness or disorder and were therefore supported by caregivers (Table 1). Only 36.7% of epileptics had a permanent job compared to 82.9% of healthy controls, and as many as 36.7% received a pension/retirement compared to 2.9% of the controls. The control and study groups did not differ in terms of sex, age, BMI or vitamin D supplementation.

### Biochemical analyses

In this study, the concentrations of cholesterol and triglyceride fractions were determined. The lipid profiles of patients with epilepsy did not differ significantly from those of healthy people. The average serum LDL cholesterol (LDL-Ch) level was higher in the study group, but this difference was not statistically significant (*p* = 0.270). In both groups of participants, the LDL-Ch level was higher than that recommended by the current guidelines for the management of dyslipidemia in the European population [24] (Table 2).

**Table 2:** Serum lipid concentration in the study group and in the healthy control

	Study Group *N* = 60	Healthy Control *N* = 70	*P* value
	Mean ± SD	Mean ± SD	
TC [mg/dL]	192.60 ± 42.16	193.43 ± 38.46	0.754
LDL -C [mg/dL]	128.30 ± 44.55	117.71 ± 35.98	0.270
HDL- C [mg/dL]	54.48 ± 14.09	56.44 ± 13.08	0.446
TG [mg/dL]	109.20 ± 72.21	101.74 ± 53.59	0.939

*TC* total cholesterol, *LDL* low density lipoprotein, *LDL-C* LDL cholesterol, *HDL* high density lipoprotein, *HDL-C* HDL cholesterol, *TG* triglycerides, *N* number, *SD* standard deviation

### Body composition

During the study, an analysis of body composition was performed using the bioelectric impedance method [25]. Generally, parameters such as protein content, minerals, water content, fat free mass (FFM), muscle mass, waist-to-hip ratio (WHR), and visceral fat did not differ significantly between the epilepsy participants and the control group (Table 3); however, in epileptic men, a significantly higher percentage of body fat (PBF) was found (*p* = 0.0142). A similar tendency was observed with regard to visceral fat, which was 33% higher in men with epilepsy, but the difference was not statistically significant (*p* = 0.0743).

**Table 3:** Body composition parameters in the study and control groups

Parameter	Study Group *N* = 35	Healthy Control *N* = 47	*P* value	Study group *N* = 25	Healthy control *N* = 23	*P* value
	**Women**			**Men**		
Protein [kg] mean ± SD	8.84 ± 1.09	9.29 ± 1.14	0.0754	12.44 ± 2.16	13.07 ± 1.61	0.2612
Minerals [kg] mean ± SD	3.22 ± 0.46	3.36 ± 0.41	0.1505	4.31 ± 0.67	4.44 ± 0.62	0.4900
Total Body Water [L] mean ± SD	33.03 ± 3.96	34.53 ± 4.12	0.1013	46.18 ± 7.96	48.09 ± 6	0.3560
Fat Free Mass [kg] mean ± SD	45.1 ± 5.5	47.19 ± 5.65	0.0649	62.93 ± 10.78	65.6 ± 8.19	0.3422
Percent Body Fat [%] mean ± SD	32.1 ± 8.98	30.71 ± 7.37	0.4440	27.65 ± 7.8	22.2 ± 6.94	0.0142
Skeletal Muscle Mass [kg] mean ± SD	24.76 ± 3.25	25.95 ± 3.47	0.1186	35.38 ± 6.45	37.38 ± 4.84	0.2337
Waist-to- Hip Ratio mean ± SD	0.9 ± 0.06	0.9 ± 0.07	1.0000	0.97 ± 0.11	0.95 ± 0.08	0.3914
Visceral Fat Level mean ± SD	10.06 ± 5.02	9.49 ± 4.42	0.5873	11.12 ± 5.79	8.35 ± 4.59	0.0743

*N* number, *SD* standard deviation

### Dietary assessment

Compared to controls, epileptic participants consumed cottage cheese (*p* < 0.001), fruit (*p* = 0.005), pulses (*p <* 0.001), nuts and seeds (*p* = 0.009), sugar, honey and sweets (*p* = 0.015), coffee (*p* = 0.010), and alcohol significantly less often (*p <* 0.001) (Table 4). In contrast, they consumed sugar-sweetened soda more often (*p* = 0.022).

**Table 4:** Food frequency consumption according to ranks, determined in the Chi^2 test

Food	Study Group Mean ± SD	Healthy Control Mean ± SD	*P* value
	rank	rank	
White bread	3.62 ± 1.56	3.57 ± 1.6	0.444
Wholemeal bread	3.26 ± 1.4	3.67 ± 1.31	0.096
Fine grained groats, white rice	2.62 ± 1.1	2.74 ± 0.97	0.726
Coarse grained groats, brown rice	2.22 ± 1.2	2.46 ± 1.07	0.300
Milk, yoghurt, kefir	3.48 ± 1.43	4.11 ± 1.32	0.108
Cottage cheese	2.13 ± 1.11	2.98 ± 1.04	< 0.001
Cheeses and processed cheeses	2.81 ± 1.17	3.18 ± 1.19	0.321
Poultry meat and sausages	3.43 ± 1.25	3.67 ± 1.11	0.546
Pork meat and sausages	3.85 ± 1.21	3.9 ± 1.27	0.688
Fish	2.4 ± 1.03	2.44 ± 0.71	0.050
Eggs	3.16 ± 1.12	3.24 ± 0.94	0.074
Raw vegetables	3.66 ± 1.53	4.3 ± 1.16	0.083
Fruit	3.7 ± 1.6	4.46 ± 1.0	0.005
Pulses	2.02 ± 1.11	2.6 ± 0.92	< 0.001
Nuts, seeds	2.5 ± 1.4	2.98 ± 1.22	0.009
Sugar, honey, sweets	3.4 ± 1.5	3.76 ± 1.4	0.015
Fruit juices	2.23 ± 1.28	2.41 ± 1.16	0.217
Sugar-sweetened soda	2.6 ± 1.7	1.87 ± 1.07	0.022
Coffee	3.6 ± 1.97	4.73 ± 1.54	0.010
Beer	1.48 ± 0.85	2.36 ± 1.19	< 0.001
Wine	1.3 ± 0.53	1.91 ± 0.88	< 0.001
Spirits	1.23 ± 0.64	1.59 ± 0.67	< 0.001
Fast food	1.52 ± 0.73	1.69 ± 0.77	0.551

*SD* standard deviation

**Fig. 2:**
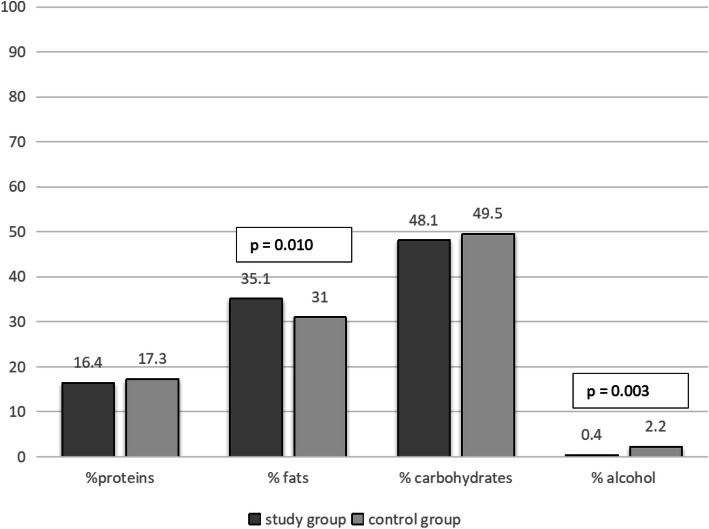
Percentage of energy from macronutrients and alcohol in the total energy supply in epileptic and healthy participants

Energy and macro- and micronutrients were calculated on the basis of 24-h dietary records from the three consecutive days preceding the study. The mean energy of the study group (1777 ± 557 kcal/d) did not differ from that of the control group (1723 ± 534 kcal/d). In epilepsy patients, a significantly higher percentage of energy from fat (35.08 ± 9.11%) was found compared to healthy subjects (30.96 ± 7.35%) (*p* = 0.010). The percentage of macronutrients in the energy supply for both groups is shown in Fig. 2.

Table 5 shows a comparison of select nutrient intake in the study group and control group. The patients with epilepsy consumed significantly less vitamin B3 in their diet than the controls (*p* = 0.047). In both groups, however, the mean values were in line with the estimated average requirement (EAR), which is set to meet the requirements for 50% of a given population. In the study group, more people (as many as 25%) did not meet the EAR, while among healthy volunteers, it was only 7%. The same was the case with vitamin C, although in both groups, the average intake was consistent with the EAR. However, in the study group, the intake was significantly lower (*p* = 0.005); every other person did not consume enough vitamin C. Moreover, significantly lower potassium intake was found in people with epilepsy than in the control group (*p* = 0.022), which was not within the range of sufficient consumption for the Polish population. Almost three-fourths of patients with epilepsy presented a below average intake (AI) of potassium in their diet. Moreover, in the study group, sodium intake was higher than that in the control group, and in both groups, more than 200% AI for this element was consumed. Individuals in both the study group (83% < AI) and the control group (57% < AI) showed insufficient calcium intake. In addition, as many as 96% of the subjects from the study group and ~ 97% of the controls did not consume enough vitamin D in their diet. The intake of saturated, monounsaturated, and polyunsaturated fatty acids and cholesterol was higher in the study group than in healthy individuals, but this difference was not statistically significant (*p* = 0.077; *p* = 0.116; *p* = 0.262, respectively).

**Table 5:** Dietary intake of selected vitamins, minerals, fatty acids, and cholesterol in the study and control groups

	Study Group Mean ± SD (% deficient)	Healthy Control Mean ± SD (% deficient)	*P* value
Vitamin B1 [mg]	1.19 ± 0.47 (120)	1.24 ± 0.51 (128.7)	0.538
<EAR (%)	33.3	34.2	
Vitamin B2 [mg]	1.52 ± 0.51 (155.64)	1.58 ± 0.62 (164.49)	0.525
<EAR (%)	13.3	10	
Vitamin B3 [mg]	18,2 ± 9,92 (158,26)	20,06 ± 8,05 (176,78)	0.047
<EAR (%)	25,0	7,14	
Vitamin B6 [mg]	1.88 ± 1.2 (166.04)	1.89 ± 0.88 (165.83)	0.293
<EAR (%)	16.7	17.14	
Folic acid [μg]	244.23 ± 85.18 (76.3)	270.23 ± 87.09 (84.45)	0.081
<EAR (%)	80	77.14	
Vitamin B12 [μg]	4.28 ± 3.9 (213.83)	3.58 ± 2.86 (179.03)	0.304
<EAR (%)	21.7	22.8	
Vitamin A [μg]	1113.5 ± 966.25 (202.55)	995.4 ± 729.2 (183.27)	0.772
<EAR (%)	20	25.7	
Vitamin C [mg]	83.06 ± 63.29 (124.85)	122.15 ± 92.52 (189.09)	0.005
<EAR (%)	50	31.4	
Vitamin D [μg]	3.79 ± 5.45 (25.26)	2.85 ± 2,.56 (19)	0.469
<AI (%)	96	98.6	
Sodium [mg]	3524.27 ± 1313.5 (234.95)	3096.7 ± 1091.4 (206.45)	0.046
<AI (%)	1.6	1.4	
Potassium [mg]	2939.11 ± 1033.13 (83.97)	3335.05 ± 1077.65 (95.29)	0.022
<AI (%)	73.3	55.7	
Calcium [mg]	575.2 ± 281.55 (55.65)	623.71 ± 252.72 (70.98)	0.211
<EAR (%)	83.3	57.14	
Phosphorus [mg]	1170.84 ± 410.65 (200.96)	1225.62 ± 362.04 (211.31)	0.338
<EAR (%)	5	1.4	
Magnesium [mg]	282.57 ± 94.81 (95.75)	308.66 ± 104.48 (107.96)	0.258
<EAR (%)	51.6	51.4	
Iron [mg]	10.6 ± 3.41 (158.24)	11.11 ± 3.18 (159.36)	0.245
<EAR (%)	16.6	12.85	
Zinc [mg]	9.57 ± 3.22 (121.61)	9.66 ± 2.87 (126.79)	0.698
<EAR (%)	33.3	24.2	
Iodine [μg]	117.03 ± 50.92 (123.19)	113.11 ± 46.88 (119.06)	0.671
<EAR (%)	33.3	40	
SFA [g]	25.41 ± 10.79	21.97 ± 9.94	0.077
MUFA [g]	30.05 ± 16.7	25.1 ± 10.6	0.116
PUFA [g]	10.04 ± 6.09	8.86 ± 3.8	0.262
Cholesterol [mg]	287.22 ± 118.1	270.97 ± 148.11	0.241

*SD* standard deviation, *EAR* Estimated Average Requirement, *AI* Average Intake, *SFA* short chain fatty acids, *MUFA* monounsaturated fatty acids, *PUFA* polyunsaturated fatty acids

### Serum 25(OH)D3 concentration

The median serum concentration of 25(OH)D3 in epilepsy patients was 77.1 (63.81–89.02) nmol/L, which was significantly lower than that in healthy subjects (90.228 (77.41–102.67) nmol/L) (*p* = 0.000074). In both groups, the mean results were within the recommended range (> 75 nmol/L) [26]. Additionally, the serum concentration of 25(OH)D3 was analyzed according to sex and age. Women in the study group had significantly lower concentrations of 25(OH)D3 than those in the healthy group (*p* = 0.000693), although there were no significant observed differences in men (*p* = 0.062509). Regardless of age, the serum concentration of 25(OH)D3 in the study group was lower than that in the healthy group (*p* = 0.000409 in the group < 35 years and *p* = 0.039658 in the group ≥35 years) (Table 6).

**Table 6:** Serum concentration of 25-hydroxyvitamin D3^a^

25-hydroxyvitamin D3 [nmol/L]	Study Group ***N =*** 60	Healthy Control ***N =*** 70	***P*** value
	Median (P25-P75)	Median (P25-P75)	
Median	77.1 (63.81–89.02)	90.228 (77.41–102.67)	0.000074^a^
Women	79.58 (64.03–87.6)	89.93 (78.44–103.48)	0.000693^a^
Men	72.018 (63.59–94.03)	92.22 (72.60–102.04)	0.062509
Age			
< 35	75.794 (63.59–84.59)	92.088 (79.207–102.36)	0.000409^a^
≥ 35	78.426 (64.74–94.03)	85.623 (74.54–103.48)	0.039658^a^

^a^Adjusted for season

*N* number, *P25* 25th percentile, *P75* 75th percentile

### Physical activity

The data collected from the participants on physical activity are shown in Table 7. Epileptic participants performed significantly less moderate (*p* = 0.010) or intense physical activity (*p* = 0.009) than the healthy subjects. They also spent significantly more hours sitting during the day (*p* = 0.022) and less time spent walking (*p* = 0.025). Generally, the overall level of physical activity (MET-min per week) was insignificantly lower in epileptic individuals (*p* = 0.139); however, according to the IPAQ’s interpretation criteria, the level of physical activity was sufficient in both groups.

**Table 7:** Physical activity (PA) assessed with the International Physical Activity Questionnaire – Short Form (IPAQ-S)

Parameter IPAQ-S	Study Group Mean ± SD	Healthy Control Mean ± SD	*P* value
Vigorous physical activities (days per week)	1.12 ± 2.02	1.77 ± 2.02	0.009
Moderate physical activities (days per week)	1.78 ± 2.65	2.33 ± 2.38	0.010
Walk for at least 10 min at a time (days per week)	5.40 ± 2.27	5.89 ± 2.03	0.103
Time spent walking (minutes per day)	104.1 ± 135.9	136.4 ± 145.7	0.025
Time spent sitting (hours per day)	6.88 ± 2.70	5.67 ± 3.03	0.022
Level of PA (MET-min/week)	4038 ± 6126	5801 ± 7222	0.139

*SD* standard deviation, *MET* metabolic equivalents of task

The potential relationship between serum 25(OH)D3 concentration and physical activity was studied, and the results are shown in Table 8. The study group showed a significant, weak positive correlation between the time spent walking during the day and overall physical activity (expressed in MET-min/week) and vitamin D3 concentration (*p* = 0.031 and *p* = 0.018, respectively). There was also a significant negative correlation between the number of hours spent sitting per day and 25(OH)D3 (*p* = 0.047). No such correlations were found in the control group.

**Table 8:** Spearman rank correlations between the serum 25(OH)D3 concentration and physical activity according to the International Physical Activity Questionnaire – Short Form (IPAQ-S) parameters

	Study Group Correlation *R* value (p)	Healthy Control Correlation *R* value (p)
Vigorous PA (days per week)	0.114 (0.387)	0.023 (0.847)
Moderate PA (days per week)	0.163 (0.214)	−0.069 (0.569)
Walk for at least 10 min at a time (days per week)	0.244 (0.060)	−0.071 (0.562)
Time spent walking (minutes per day)	0.279 (0.031)	− 0.032 (0.792)
Time spent sitting (hours per day)	−0.257 (0.047)	− 0.156 (0.198)
Level of PA (MET-min/week)	0.305 (0.018)	−0.04 (0.74)

*PA* physical activity, *MET* metabolic equivalents of task

## Discussion

Epileptic people are a difficult group to study due to frequent mental, behavioral and social problems [17, 27]. The current studies on dietary behavior and lifestyle habits in this group of patients are limited [9, 12, 17, 28, 29] and need to be expanded. For this research, recruitment took place during the 2016–2019 period through various information channels. A total of 70 epilepsy patients were enrolled in the study, which was quite a good result considering the reluctance of epileptic patients to participate in scientific research [30].

Studies have shown that epileptic patients have a higher risk of comorbidities, including dyslipidemia [10, 31], which is a risk factor for cardiovascular disease. One of the risk factors for dyslipidemia can be antiepileptic treatment, but it cannot be clearly determined which kind of medication (drugs and drug combinations) has the greatest impact [32]. In some studies, monotherapy with older-generation drugs such as carbamazepine, valproic acid, and phenytoin was associated with an increase in cardiovascular risk [33]. In contrast, a study by Vicanco-Hidalgo et al. [34] concluded that despite a higher percentage of dyslipidemia in epileptic patients, they do not have greater cardiovascular risk. In the above study, compared to healthy individuals, those with epilepsy had lower rates of hypertension and diabetes [34], illustrating the need for better assessment of epileptic patients prior to statin treatment. In our study, patients were treated with various antiepileptic drugs, both in mono- and polytherapy. Although we found elevated levels of LDL cholesterol in the blood serum of epileptic patients compared to the controls, the results were not statistically significant. No significant differences were found between these groups with respect to other lipids. These and other findings suggest that epilepsy treatment may not be the only factor behind dyslipidemia. Other factors, such as dietary behavior and lifestyle, can contribute to disorders of lipid metabolism. Kim et al. [28] analyzed the effect of lifestyle modification and the use of statins on the reduction in vascular risk among patients with epilepsy. Pharmacotherapy was found to be more effective, but lifestyle changes also had positive effects.

There are very few studies in the literature on nutritional status and dietary habits in epilepsy. One of them, the California Health Interview Survey, concluded that eating behaviors and physical fitness levels among epileptic individuals are similar to those of healthy people [29]. In contrast, some evidence shows that epilepsy may increase overweight and obesity, as well as the risk of cardiovascular diseases [10]. The percentage of overweight and obesity measured in our study was 53.4% for epilepsy patients and 52.8% for healthy controls, which was similar to another study reporting a percentage of 55.2% [35]. In addition, we analyzed body composition using electrical bioimpedance. A significantly higher percentage of body fat (PBF) was found among epileptic men than among healthy men (27.65% vs. 22.2%, respectively). However, no significant differences were found among women. There is a lack of research on body composition in epilepsy. In another study, patients treated with antiepileptic drugs had a PBF similar to the controls [36]. According to the World Health Organization (WHO), abdominal obesity is a risk factor for metabolic diseases [37]. Overall, the average waist-to-hip ratio (WHR) in this study, regardless of the prevalence of epilepsy, was above the normal range for both women and men. Due to these results and the associated risk of metabolic diseases, it is reasonable to pay attention to the dietary behavior of patients with epilepsy [38]. Some studies have shown high carbohydrate and protein intake in epilepsy as well as low fat intake, including polyunsaturated fatty acids [12]. In contrast to these results, our research reveals a different tendency. While both carbohydrate and protein intake were at the recommended levels, more fat was consumed by the epilepsy patients than by the controls, yet the intake of polyunsaturated fatty acids (PUFAs) was similar across both groups of participants. Compared to another study [12], the average cholesterol intake of epilepsy patients in our study was in the normal range for both men and women.

The frequency of food consumption was further analyzed, and the results revealed that patients with epilepsy showed less favorable eating behavior than healthy people. They consumed plant-based products such as fruit, pulses, seeds, and nuts, which are sources of vitamins, minerals, and dietary fiber, significantly less frequently. These food products are recommended for the prevention of cardiovascular risk [39]. However, in our study epilepsy patients showed less frequent consumption of vegetables and fruits and sugar sweetened soda, but consumed legumes more often than the epileptic individuals of the California Health Interview Survey [29]. Some countries have formulated dietary guidelines for epilepsy patients. For example, in the UK, the dietary recommendations emphasize a balanced diet providing all macronutrients, with a particular emphasis on vegetables and fruits [40]. Attention is also focused on foods that provide complex carbohydrates, which raise blood glucose levels more slowly and for longer, allowing for a longer sense of satiety after a meal. Similarly, the dietary recommendations for epilepsy patients in the US are mainly based on the elimination of monosaccharides and food products with high glycemic indexes from the diet while promoting a balanced, varied diet and an appropriate amount and quality of fluids [41]. In our study, epileptic participants consumed fewer dairy products, including cottage cheese, which is a source of easily absorbable calcium that is important for bone health. In addition, fish and eggs, which are food sources of vitamin D, were consumed less frequently, but this difference was insignificant. It is worth noting that patients with epilepsy also showed a lower serum concentration of 25(OH)D3. In this study, epilepsy patients were more likely to consume sugar-sweetened soda, which, due to the content of sugar, preservatives, and dyes, are considered less beneficial to health [42].

The intake of stimulants such as coffee and alcohol was analyzed in this study, and the patients with epilepsy were found to consume coffee less often than healthy individuals. There are many indications that regular coffee consumption may exacerbate seizures in some patients due to its effect on the central nervous system [43, 44]. Thus far, the role of caffeine in the control of epilepsy is unclear. Studies on animal models suggest that depending on the dose and length of caffeine intake, it may have both positive and negative effects on seizure control [45, 46]. Moreover, caffeine may reduce the effectiveness of some anticonvulsant drugs, mainly topiramate [47]. Some studies have indicated the need to reduce caffeinated beverages due to the possibility of increasing the frequency of epileptic seizures and reducing the effectiveness of anticonvulsant drugs. It should be noted that caffeine is not currently considered a seizure-inducing factor [48].

Epileptic participants in this study consumed alcohol less frequently than the control group, which is consistent with the findings of Elliot et al. [29]. A study by Hinnell et al. [49] found that patients with epilepsy consumed alcohol less frequently than the general population; however, one-fourth of them disclosed regular consumption of alcoholic beverages. In another study, patients with epilepsy reported addictive drinking less frequently than healthy people [50]. The influence of alcohol consumption on the occurrence of epileptic seizures has been the subject of many studies. Some of them suggest that moderate alcohol consumption does not increase the frequency of seizures, while others show the possibility of intensifying epileptic seizures due to various mechanisms of action, e.g., effects on neurotransmission and metabolic changes [51]. Hamerle et al. [52] found a negative impact of significant amounts of alcohol on epileptic patients but concluded that moderate consumption seems safe for most patients. This scientific research has been reflected in the dietary recommendations for epileptic patients. The recommendations for patients in Australia point to the negative consequences of alcohol consumption, especially larger quantities, and the need to consult a physician based on the drug regimen prescribed [53]. It is important to note that alcohol may interact with anticonvulsant drugs, reduce their effectiveness, intensify side effects, and increase the risk of seizures caused by sleep disturbances and reduced sleep quality [54].

The analysis of vitamin and mineral intake showed that most (over 80%) nutrient deficiencies were in folic acid, vitamin D, and calcium. This is particularly important due to the role of B vitamins, including folic acid and vitamin D, in the proper development and functioning of the nervous system [55]. Moreover, vitamin D and calcium are responsible for bone and mental health, and patients treated with antiepileptic drugs are particularly vulnerable to osteoporosis and depression [56]. Others have shown low calcium intake among patients with epilepsy [57]. Our study also found significantly lower vitamin C and potassium intake and higher sodium intake among epileptics. In contrast to supplementation studies, meta-analyses of prospective studies found that higher dietary intakes of vitamin C and potassium were associated with a reduced risk of cardiovascular disease [58, 59]. In contrast, increased sodium intake was associated with higher mortality due to CVD [60]. The intake of minerals and vitamins in epilepsy has rarely been studied. Therefore, there is a need for more research that would allow for the formulation of dietary recommendations for this group of patients that consider potential deficiencies in these nutrients.

The role of vitamin D in the etiology and course of epilepsy is still not clearly understood; however, in the 1990s, a higher birth rate of children with epilepsy was observed in the winter period [61]. It has also been noted that in patients with epilepsy, both the severity of epileptic seizures, their frequency, and the number of sudden unexpected deaths occur during the winter period, when insolation is limited [62]. In animal models, vitamin D may reduce the severity of chemically induced seizures and increase the anticonvulsant effect of some antiepileptic drugs [63, 64]. However, studies assessing serum concentrations of 25(OH)D3 in epileptic patients are still scarce. In a study conducted in India, lower average serum concentrations of vitamin D were observed compared to our results [65]. Although the territory of India is characterized by high sunshine, clothing covers most of the body, limiting skin synthesis of vitamin D. Cultural differences and skin pigmentation are also important. In the above study, no significant differences in terms of sex or between the study and control groups were found. A recent literature review found that vitamin D deficiency affects the entire European population [66]. Other European studies have reported significantly lower serum concentrations of vitamin D in epilepsy patients and lower seizure rates after vitamin D supplementation [67]. However, it should be noted that this study was conducted on a small group of 13 patients with epilepsy. A larger US study found frequent vitamin D deficiency in patients with epilepsy [6]. The average serum concentration of 25-hydroxyvitamin D in the epileptic population was lower than that in the general population.

A significant portion of the research on epilepsy has focused on the influence of antiepileptic drugs, which cause a decrease in the serum vitamin D concentration, negatively affecting the skeletal system [68]. When considering the possible influence of drugs on vitamin D, one should note the frequent co-occurrence with epilepsy of other disorders, including mental disorders. Mental disorders occurring during the interictal period are the most common problem in epilepsy patients. Dementia syndrome, interictal psychosis, and affective disorders such as mania or depression among interictal psychiatric disorders can be distinguished. Patients with mental disorders are particularly at risk of vitamin D deficiency, especially those treated with clozapine, as well as people with low physical activity and poor eating habits [69].

Physical activity is a lifestyle element that is important for maintaining health. Although participants with epilepsy as well as healthy participants in the study were moderately active, those with epilepsy spent more time during the day sitting and less often performed intensive and moderate physical activity during the week, consistent with the findings of others [70]. This lower level of physical activity may be due to the low occupational activity of epileptics compared to healthy people (36.7% vs. 82.9%, respectively). The latest meta-analysis of population studies shows that further research is needed before specific recommendations on physical activity in people with epilepsy can be formulated [70]. Among the patients with epilepsy, but not those in in the control group, a positive relationship was found between the level of physical activity and serum concentration of vitamin D3. Vitamin D is mainly synthesized by sunlight, so outdoor physical activity can increase the concentration of this vitamin in serum. Some studies, however, show that any type of physical activity, including indoor activity, may increase the synthesis of vitamin D [71], which was also found in our study. Although there were no significant differences in the level of physical activity between the study group, in which the serum vitamin D concentration was significantly lower, and healthy controls, the level of physical activity in both groups was estimated to be moderate. However, the participants with epilepsy spent much less time walking and more time sitting. Moreover, in epileptics, a weak positive correlation was observed between serum vitamin D concentration and time spent walking and between serum vitamin D and overall physical activity. There was also a weak negative correlation between vitamin D concentration and the time spent sitting. Unfortunately, numerous studies have indicated that people with epilepsy are less physically active and less willing to participate in sports activities [72]. In general, fewer people with epilepsy perform physical activity at the recommended level [45]. Importantly, other studies have addressed the potentially beneficial role of physical activity in improving the cognitive function of patients with epilepsy [73] and quality of life [74]. Scientific evidence has indicated that it is reasonable to educate patients with epilepsy about their lifestyle habits. Despite the proven role of physical activity in epilepsy, as shown by research results, recommendations for physical activity are not uniform. In some countries, epilepsy patient associations promote recommendations for physical activity [75]; however, there are no general guidelines. When making such recommendations, factors such as the type of epilepsy, seizure control, type of treatment, and safety of various forms of physical activity should be taken into account [16].

This study has several limitations that should be mentioned. It assessed various aspects of nutritional behavior and lifestyle, as well as biochemical and anthropometric parameters, which is a strength of this study but can also be a burden on participants. Therefore, a smaller number of respondents volunteered to take part in the survey than initially assumed. On the other hand, the data obtained during this research were complete for each participant. Another limitation is related to the data on dietary behavior and physical activity, which were based on self-reports and may therefore be biased.

## Conclusions

Our study found that patients with epilepsy have several lifestyle-related risk factors that predispose them to cardiovascular diseases, thus confirming the results of other studies. They were more sedentary, had worse body composition, higher LDL, lower vitamin D and less favorable dietary behavior than healthy people. In conclusion, the results of this study indicate that several behavior-related habits, which may predispose epileptic people to cardiovascular diseases, need to be improved. For this reason, patients with epilepsy should be provided with more comprehensive medical care, including advice on nutrition and physical activity.

## Supplementary Information

**Additional file 1. **STROBE Statement.

## Data Availability

The datasets used and analysed during the current study are available from the corresponding author on reasonable request.

## Abbreviations

AI: Average intake
BHT: Butylated hydroxytoluene
BM: Body mass
BMI: Body mass index
CVD: Cardiovascular disease
DAD: Diode array detector
EAR: Estimated average requirement
HDL: High density lipoprotein
HPLC: High pressure liquid chromatography
IPAQ: International Physical Activity Questionnaire
IPAQ-S: IPAQ short form
LBM: Lean body mass
LDL: Low density lipoprotein
LDL-Ch: LDL cholesterol
MET: Metabolic equivalent of task
PA: Physical activity
PBF: Percentage body fat
PWC: Percentage water content
SMM: Skeletal muscle mass
25(OH)D3: 25-hydroxyvitamin D3
